# Perceptual History Acts in World-Centred Coordinates

**DOI:** 10.1177/20416695211029301

**Published:** 2021-10-09

**Authors:** Kyriaki Mikellidou, Guido Marco Cicchini, David C. Burr

**Affiliations:** Department of Psychology, University of Cyprus, Nicosia, Cyprus; Centre for Applied Neuroscience, Nicosia, Cyprus; Department of Neuroscience, University of Florence, Florence, Italy; Institute of Neuroscience, National Research Council, Pisa, Italy; Institute of Neuroscience, National Research Council, Pisa, Italy; Department of Neuroscience, University of Florence, Florence, Italy; School of Psychology, University of Sydney, Sydney, Australia

**Keywords:** serial dependence, coordinate frames, head movement, predictive coding, perception

## Abstract

Serial dependence effects have been observed using a variety of stimuli and tasks, revealing that the recent past can bias current percepts, leading to increased similarity between two. The aim of this study is to determine whether this temporal integration occurs in egocentric or allocentric coordinates. We asked participants to perform an orientation reproduction task using grating stimuli while the head was kept at a fixed position, or after a 40° yaw rotation between trials, from left (−20°) to right (+20°), putting the egocentric and allocentric cues in conflict. Under these conditions, allocentric cues prevailed.

## Introduction

Perception depends not only on the stimuli impinging on our senses but is strongly conditioned by expectations and past perceptual experience. Many perceptual properties—such as orientation, numerosity and face perception—are systematically biased towards the recent perceptual experience (Cicchini et al., 2014, 2017; Fischer & Whitney, 2014; Liberman et al., 2014). This effect, known as *serial dependence,* probably reflects an optimisation strategy, where perceptual systems take advantage of temporal redundancies (the relative stability of the world) to improve signal to noise ratios and hence efficiency (Cicchini et al., 2018). Much evidence suggests that serial dependence acts directly within perceptual circuitry, at early stages of information processing (Cicchini et al., 2021), including monaural auditory circuits (Hao Tam Ho et al., 2019) and primary visual cortex (V1; St. John-Saaltink et al., 2016). In this study, we show that serial dependence for orientation judgements is spatially selective in external, not retinal coordinates, reinforcing the notion that it is driven by the temporal continuity of the external world.

## Methods

We measured serial dependence for orientation perception, with participants periodically tilting their heads from side to side between trials to dissociate retinotopic from spatiotopic representations.

### Stimuli and Apparatus

**Figure 1.**
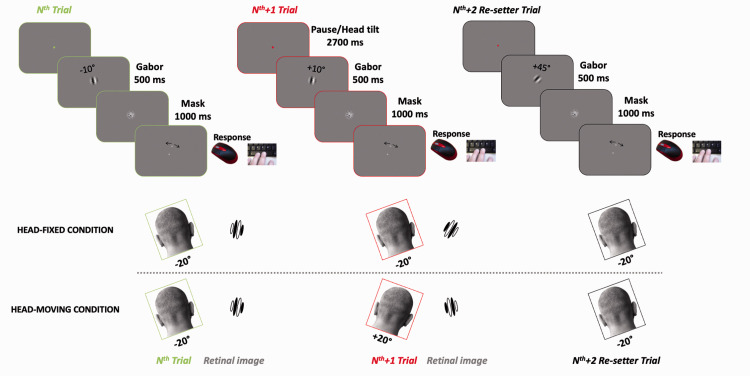
Timeline of experiment for the head-fixed and head rotating conditions, showing three consecutive trials. Trial sequence was designed so that the second trial of the triplet contained an orientation change of 20° (in this example 20° clock-wise [CW]). This is paired with a head rotation of the same direction but twice in amplitude (40° CW). The overall result is that the retinal image (shown in the inset only for the crucial trials) rotates in the opposite direction (20° counter-cockwise [CCW]).

Figure 1 illustrates the experimental setup and timeline. Each trial began with the fixation point whose colour signalled which side the observer should position their head. After a 2,700 milliseconds pause, sufficient to complete the yaw rotation (Mikellidou et al., 2016), a grating patch was presented centrally (spatial frequency 0.3 cpd, contrast 25%, 500 milliseconds, 3.2° full-width half-height), followed by a mask (random noise filtered at 0.3 cpd, contrast 50%, 1,000 milliseconds). Observers reproduced the perceived orientation of the patch (always with the head tilted) by setting the orientation of a mouse-controlled virtual line marked at its ends by two small circles (diameter 0.2°). Participants confirmed their choice with the space bar and the reproduction cursor disappeared.

In a typical *head-fixed paradigm* (Figure 1—top rows), egocentric and allocentric coordinate frames are the same. To dissociate between the two, we asked observers to rotate their heads around the yaw axis, from resting on the left plate (approx. −20° from vertical) to the right plate (approx. +20° from vertical) from one trial to another (bottom row). When the head rotates by 40° and the stimulus rotates 20° in same direction, the rotation in allocentric coordinates is +20° and in egocentric (retinotopic) coordinates −20°, equal and opposite to that in allocentric coordinates. These trials were crucial to distinguish between the coordinate frames of the effects.

We concatenated triplets of trials in which the first two stimuli follow the above rule, and the third stimulus acts as a reset, with its orientation chosen either 35° or 55° away from the preceding stimuli. Successive triplets started at least 45° away from the preceding trial leading to stimulus sequences where the critical pair of trials differed in orientation by 20° while the differences between the remainder of the possible pairs most often (>90% of the trials) exceeded 30°.

Only data from the crucial trials were isolated and analysed. Adjustment responses which took longer than 3 seconds or created an error in excess of 35° were discarded (1.1% of trials). As orientation reproduction may exhibit biases due to attraction towards diagonals (or repulsion from cardinals; de Gardelle et al., 2010; Jastrow, 1892; Taylor & Bays, 2018), we first removed subjective orientation-specific biases by subtracting the average response to that orientation in noncritical trials (where the intertrial difference exceeded 35°). We then estimated the weight of the previous stimulus by dividing the specific error to a given trial by the orientation difference with the previous trial, pooling over all orientations. To estimate the egocentric and allocentric component of serial dependence, we collected on each participant two sessions in fixed head condition and two in alternating condition (48 trials in each session-96 in total for each condition).

 Stimuli were generated under MATLAB version 7.6 using Psychtoolbox routines (Brainard, 1997; Kleiner et al., 2007; Pelli, 1997) and presented on a 23-inches LCD monitor (52° × 29°) with 1,920 × 1,080 resolution at a refresh rate of 60 Hz and mean luminance of 38 cd/m^2^. Observers viewed the stimuli binocularly from a distance of 57 cm.

### Participants

Sixteen participants were initially screened with the head-stationary condition. Those who showed significant positive serial dependence when the head was still were asked to complete the second part of the experiment, with the head rotating between the left and right head rests as described earlier. Eight participants (six females: age range 23–40 years old) completed both conditions. All observers except authors K.M and G.M.C. were naive to the objective of the experiment, and all had normal or corrected-to-normal vision. Experimental procedures were approved by the regional ethics committee (*Comitato Etico Pediatrico Regionale-Azienda Ospedaliero-Universitaria Meyer*, Florence) and are in line with the Declaration of Helsinki. All participants gave informed written consent.

Eight out of 16 participants showed no positive serial dependence. It is unclear why only half had positive serial effects under these conditions, but several reasons are possible. First, many studies (Bliss et al., 2017; Turbett et al., 2019; Zhang & Alais, 2020) have reported that the magnitude of serial dependence varies considerably between participants, from strong positive effects to clear negative effects. In the current paradigm, the stimulus was presented centrally (necessary to dissociate orientation but not spatial position), rather than peripherally, as in many serial dependence studies of orientation (Cicchini et al., 2017; Fischer & Whitney, 2014). In addition, making psychophysical judgements with the head tilted was somewhat uncomfortable, which could have added to the noisiness of the judgements, obscuring the effect. But whatever the reasons for the variability in sign and magnitude of serial dependence effects, we were interested only in the spatial coordinate system for assimilative serial dependence: Retinotopy for orientation adaptation has been addressed extensively elsewhere (Knapen et al., 2010; Melcher, 2005).

## Results

**Figure 2.**
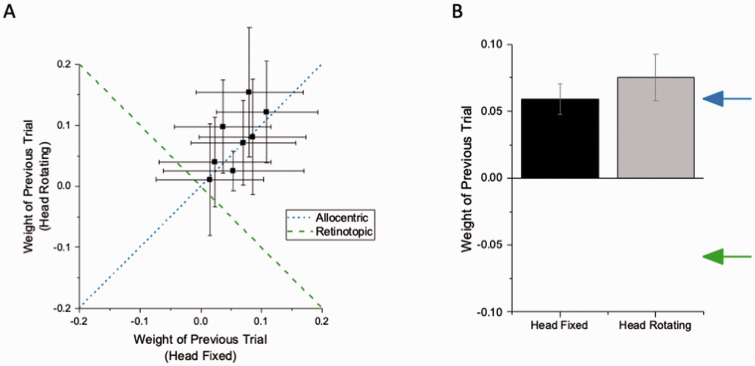
A: Individual data points for serial dependence in the head-fixed condition (where allocentric and retinotopic effects coincide) and head rotating condition (where allocentric and retinotopic effects are in opposing directions). Data clearly follow allocentric coordinates. B: Average bar plots for the head-fixed (black) and head moving (grey) conditions, showing that serial dependence carries a strong allocentric component. Blue arrow indicates allocentric predictions; green arrow indicates retinotopic predictions.

Participants reproduced the orientation of briefly presented grating patches, while tilting their heads alternately between −20° (left) to +20° (right) between trials, as well as with their head stationary, resting on one of the two head rests, as described earlier (see Figure 1A). Figure 2A shows data for all participants, plotting head-fixed against alternating-tilt conditions. If the effect depends on allocentric orientation of the stimulus, then tilting the head should make no difference to the magnitude of the effect, and the data should align with the positive (blue) diagonal. On the other hand, if the effect is egocentric (or retinotopic), the sign of the effect should invert (see methods), and the data should lie on the negative (green) diagonal. Clearly, they align with the positive diagonal. The likelihood that they do so is 1.56, compared with 0.0017 that they follow the egocentric prediction, giving a likelihood ratio (Bayes factor) > 900.

Figure 2B shows the average results as bar plots, with the allocentric and egocentric predictions shown, respectively, by blue and green arrows. The average results clearly follow the allocentric predictions, with the effects measured in the head alternating condition statistically indistinguishable from those with head stationary, with BF_10_ = 0.17: substantial evidence for H_0_ (one tail, testing that the allocentric effect is less than the head-fixed effect). The serial dependence was clearly allocentric, with no measurable effect corresponding to retinal orientation, which predicts a bias in the opposite direction.

## Discussion

Our results show that under the conditions of this experiment, where the head rotated about the yaw axis between trials to put allocentric and egocentric retinal signals in conflict, serial dependence was entirely allocentric, and as strong as when measurements were made with the head still. Our paradigm does not allow us to distinguish whether the allocentric reference frame is spatiotopic, depending on the position in space, or object-centred, depending on fixed landmarks in the surrounding environment. However, we can be certain that the reference frame is not retinotopic under these conditions.

Previous evidence for the coordinate system of serial dependence has been inconsistent. In their original study Fischer and Whitney (2014) changed fixation between trials to dissociate retinotopic from allocentric serial dependence of successive stimuli in an orientation judgement task and observed both retinotopic and allocentric tuning, with broad spatial tuning (17°). On the other hand, Collins (2019) employed large 30° saccades to dissociate the two and reported clear retinotopic effects. It is unclear how the different results can be reconciled. One possibility is that remapping of spatial information after (relatively slow) head rotations is different from after rapid saccades, leading to the differences in retinotopic and allocentric contributions. Another is that our paradigm specifically puts the two effects in conflict: When in conflict, allocentric effects may dominate. This would agree with recent results looking at allocentric motion perception (Drissi-Daoudi et al., 2020).

If serial dependence serves to aid perceptual continuity, it would need to be allocentric. The external world tends to remain relatively constant over the short term, but this is not true of the retinal image: Retinal position changes on each eye-movement, and retinal orientation changes when we tilt our heads. To exploit temporal perceptual redundancies, the system requires access to allocentric information, corresponding to external reality.

Nevertheless, the result is particularly interesting and perhaps unexpected in the light of evidence for serial dependence effects in early sensory cortex (St. John-Saaltink et al., 2016), which is commonly assumed to be retinotopically rather than allocentrically tuned. Several plausible explanations are possible for the discrepancy. One is that this evidence has recently been questioned, with recent MRI classification evidence suggesting that the primary effects in V1 are of negative aftereffects, rather than positive serial dependence (Sheehan & Serences, 2021). Another possibility is that orientation signals of remembered stimuli may be reconverted back into retinotopic coordinates and projected back to V1 to interact with incoming signals. Indeed, mounting evidence, both speculative (Pascucci et al., 2019) and direct (Cicchini et al., 2021) suggests feedback from higher areas may be one of the key mechanisms mediating serial dependence. Alternatively, even primary visual areas may display more allocentric properties than is commonly assumed. Indeed, there is good evidence for partial spatiotopy in primate V1 neurones during saccades (Trotter & Celebrini, 1999) and for allocentric space representation in early visual motion areas of humans (Crespi et al., 2011). A fourth possibility is that serial dependence operates at multiple levels, both retinotopic and allocentric, and the allocentric levels predominate (as mentioned earlier).

Our study provides clear evidence that serial dependence for orientation perception operates in allocentric coordinates, taking the inclination of the head into account. This is consistent with serial dependence reflecting the perceptual predictions, which remain constant over time in allocentric, but not egocentric coordinates.
